# Supplementary material for: Dissecting the impact of transcription factor dose on cell reprogramming heterogeneity using scTF-seq
Source: Nat Genet. 2025 Oct 3;57(10):2522–35. doi: 10.1038/s41588-025-02343-7 (PMC12513835; doi:10.1038/s41588-025-02343-7)
Supplement: Supplementary file 1 — Supplementary Notes 1–15 and Figs. 1–7. [file 41588_2025_2343_MOESM1_ESM.pdf]

nature genetics

Supplementary information https://doi.org/10.1038/s41588-025-02343-7

Dissecting the impact of transcription factor dose on cell reprogramming heterogeneity using scTF-seq

In the format provided by the authors and unedited

Table of contents

Supplementary Notes 2

Supplementary Note 1: Cell culture and differentiation.. 2

Supplementary Note 2: Lentivirus production 2

Supplementary Note 3: Lentivirus transduction 2

Supplementary Note 4: Doxycycline at 2 µg/ml induces ectopic expression near saturation level 3

Supplementary Note 5: TF-ID detection and assignment to cells 3

Supplementary Note 6: Assessment and correction of batch effects...................... 4

Supplementary Note 7: Cell cycle phase classification and adjustment 5

Supplementary Note 8: Definition of functional and non-functional cells. 5

Supplementary Note 9: Logistic model and TF classification................................. 6

Supplementary Note 10: Analysis for intolerance to loss of function 7

Supplementary Note 11: Comparison of cell cycle dynamics across TFs............... 7

Supplementary Note 12: Cell death staining.. 8

Supplementary Note 13: Customized adipocyte gene set 8

Supplementary Note 14: Housekeeping gene list for expression normalization 8

Supplementary Note 15: Cell barcode assignment for TF pair overexpression 8

Supplementary Figures 10

Supplementary Fig. 1: Doxycycline at 2 µg/ml induces ectopic expression near saturation level 11

Supplementary Fig. 2: TF-ID detection and assignment....................................... 12

Supplementary Fig. 3: Comparison across batches 14

Supplementary Fig. 4: Examples of ambiguous cycling cells and correlations between S and G2M scores 15

Supplementary Fig. 5: Identification of functional TF cells.................................... 16

Supplementary Fig. 6: Flowchart of TF categorization.. 17

Supplementary Fig. 7: Fraction of cells in default cell cycle phase....................... 19

References 20

Supplementary Notes

Supplementary Note 1: Cell culture and differentiation

Both HEK293T and C3H10T1/2 cells were maintained in basic culture medium containing high-glucose DMEM with GlutaMax and pyruvate, 10% FBS and 1x Penicillin-Streptomycin. All C3H10T1/2 cells were maintained below passage 15. All cells were placed at 37 °C and 5% CO2 in a humidified incubator. Prior to use, cells were washed with PBS, dissociated with Trypsin-EDTA (0.05% for HEK293T and 0.25% for C3H10T1/2), resuspended with basic culture medium, filtered using 40 μm strainers and counted with Trypan blue live-dead stain using a Countess automated cell counter.

Supplementary Note 2: Lentivirus production

Lentiviral packing was performed using lipofectamine 2000 according to the manufacturer's instructions. First, 10 μL of Opti-MEM and 0.375 μL of lipofectamine 2000 Transfection Reagent were thoroughly mixed. A mix of 0.075 μg lentiviral expression plasmid containing the individual TF ORF (or mCherry as control) and TF-ID of interest, 0.075 μg 3rd generation lentivirus packaging plasmid mix and 10 μL of Opti-MEM were prepared. Then, two mixes were added together and incubated for 30 min at room temperature. HEK293T cells were cultured and prepared as described above, and seeded in the individual well of 96-well plates at ~95% confluency. After incubation, the transfection mix was added to freshly seeded cells. Medium was changed 12 h after transfection. 48 h post transfection, the supernatant containing virus particles was harvested, and dead cells were removed by centrifugation at 300g for 5 min. As a control, pBOB-GFP plasmid with constitutive GFP expression was transfected.

Supplementary Note 3: Lentivirus transduction

C3H10T1/2 cells were seeded 12 h prior to transduction at 10-20% density. Transduction medium was prepared by mixing the supernatant containing lentivirus particles and the basic culture medium in a 1:1 ratio, supplemented with polybrene at a final concentration of 10 μg/mL. Plated cells were then treated with the transduction medium and centrifuged at 1300 g for 30 min at 37 °C. Medium was refreshed after 24 h. After 48 h, cells were selected using 2 μg/mL Puromycin for 48-72 h. A threshold

of 10% cell loss following puromycin selection was used to ensure a multiplicity of infection (MOI) of 2.3 or higher, established by Poisson distribution. Puromycin-resistant cells were cultured in the basic culture medium to recover for 24 to 48 h.

Supplementary Note 4: Doxycycline at 2 µg/ml induces ectopic expression near saturation level

To determine whether ectopic protein expression is saturated at 2.0 µg/ml doxycycline, two mCherry-overexpressing cell lines, each with two replicates, were generated via transduction as described in **Supplementary Note 2**, expanded and induced with doxycycline (0-4 µg/ml for 5 days). To assess the distribution of fluorescence intensities, mCherry-overexpressing cells were analyzed by flow cytometry. For this, cells were harvested by trypsinization, washed with PBS and resuspended in ice-cold PBS containing 1 µg/ml DAPI (Sigma, no. D9564). Wildtype C3H10T1/2 cells served as control. Data was acquired on a BD LSR Fortessa cell analyzer. mCherry fluorescence (Ex561nm, Em610/20nm) was measured after excluding DAPI-positive dead cells (Ex355nm, Em450/50nm). Data was analyzed using FlowJo software and R (v4.3.1). The results show that 2 µg/mL doxycycline already yielded near-saturation expression levels (**Supplementary Figure 1**), consistent with previous findings[1].

Supplementary Note 5: TF-ID detection and assignment to cells

To ensure a high TF-ID recovery rate, we implemented an additional amplification and sequencing step targeting TF-IDs and cell barcodes (**Fig. 1a**, **c**, and ***10x scRNA-seq data preprocessing and quality control*** in **Methods**), which increased the percentage of cell barcodes associated with a TF-ID from 67% to 86% (**Fig. 1d**, and **Supplementary Table 2**). Given the robust correlation between the number of reads aligning to the overexpression construct in TF-enrichment and conventional single-cell libraries and the higher accuracy provided by TF-ID enrichment (**Supplementary Fig. 2a**), we used TF-enrichment libraries to assign TFs to cells. To limit the risk of wrongly assigning TFs due to sequencing errors, only TF-IDs with a hamming distance of at least 2 nucleotides were used within the same experiment (**Supplementary Fig. 2b**). Cell barcodes detected both in the 10x and TF-ID enrichment libraries with >5 TF-ID reads were retained. In all experiments, approximately 40% of cells had reads aligning to multiple TF-IDs (**Supplementary Fig. 2c**). To remove doublets and assign a TF to

a cell (**Supplementary Fig. 2c**), cells were ranked by the proportion of their main TF-ID (percentage of reads aligning to the most abundant TF-ID) from high to low for each experiment. Knee point detection was applied to find the inflexion point of the curve using the kneepointDetection function from SamSPECTRAL (v1.46.0)[2]. Cells below the knee point (on average above 80%) or with >50 reads of the second most abundant TF-ID were considered as doublets or heavily contaminated by ambient RNAs and removed.

Supplementary Note 6: Assessment and correction of batch effects

To enable robust batch integration, each experiment included mCherry controls, adipogenic reference cells (except in batch 9), and at least six TFs that were shared between batches (**Fig. 1e**, **Supplementary Fig. 3a**, and **Supplementary Table 1**).

Data integration was performed following the guidelines of Seurat. TFs that have less than 8 cells or 5 functional cells were excluded. 2,000 highly variable features were retained for integration and data scaling. The dimensionality reduction was performed via principal component analysis (PCA) with 200 PCs for the integration of all cells or 60 PCs for the integration of control cells and functional TF cells in G1 (default or adjusted cell cycle phase, as explained in **Supplementary Note 7**, and refers to functional TF atlas or phase-adjusted functional TF atlas, respectively). Notably, even though the dose range varied between experiments (**Supplementary Fig. 3b**), both pseudobulk and single-cell expression data were highly reproducible across batches (**Supplementary Fig. 3c-h**). The observed subtle gene expression variations between batches were primarily driven by differences in TF doses and dose ranges (**Supplementary Fig. 3f**, **g**).

However, to account for the impact of potential differences in TF-ID capture efficiency between batches, we aligned the TF doses across batches using shared TFs as a reference. Particularly, doses were aligned between batches 3, 4 and 9 based on 9 overlapping TFs with high max dose in each batch (>3.5). Dose response curves were calculated for each variable gene (union of all top 1000 variable genes as determined by Seurat's default FindVariableFeatures function) per batch using R smooth.spline with parameters tol = 0.5 and knots [0, 0.5, …, max-dose]. For a range of possible scaling factors (0.5-2) for batch A, the average Euclidean distance with batch B was

calculated between dose response curves. We found that the scaling factors show high reproducibility across TFs (**Supplementary Fig. 3i**-**k**). Therefore, the scaling factor with the lowest Euclidean distance, averaged across all 9 overlapping TFs, was used for all subsequent analyses.

Supplementary Note 7: Cell cycle phase classification and adjustment

To assess the impact of TF overexpression on cell cycle dynamics, we first identified the cell cycle phase per experiment by the CellCycleScoring function from Seurat (v4.4.1)[3], using a default threshold (0) of S and G2M phase scores. A more stringent threshold (0.1) of phase scores was applied for adjusted phase partitions (**Fig. 6a**). When focusing on lineage differentiation and wanting to eliminate cell cycle confounders, the default phase was used to select G1 cells and strictly remove cells that might be preparing for entering the S phase or exiting G2/M phase. The default phase was used for downstream analyses unless specified. The more stringent classification, thereafter called the “adjusted phase”, was used when focusing on cells strictly cycling and therefore rectifying the overclassification of ambiguous cells into S/G2/M, as observed, for example, for YAP1, ATF3, and control cells with S and G2M scores between 0 and 0.1 (**Supplementary Fig. 4a, b**).

Supplementary Note 8: Definition of functional and non-functional cells.

For each TF-control pair, we measured the TF-driven transcriptomic variation by calculating the Euclidean distance of cells reprogrammed by a TF to the centroids of mCherry control cells in the space consisting of top principal component coordinates (**Supplementary Fig. 5a**-**c**). More precisely, cells of each TF in each experiment were processed per cell cycle phase (default, see **Supplementary Note 7**) together with their batch-paired mCherry-overexpressing control cells, which limits confounders of the batch effect and the cell cycle and restrains directions of the data variation. After normalization and scaling on the 2000 highly variable features as implemented in Seurat, principal component analysis (PCA) was performed with 10 principal components (PCs) (**Supplementary Fig. 5a**). In the PCA space, derived from the significant PCs, three centroids were delineated for confluent, non-confluent, and all control cells to mitigate biases due to variation among control cells and their confluency. To prioritize the magnitude rather than the direction of variation, the Euclidean Distance between individual cells and the three designated centroids of the

control cells were computed. We observed that the distance between certain TF cells and the centroids of control cells is negligible (**Supplementary Fig. 5b**), allowing us to define a threshold to categorize “non-functional” versus “functional” TF cells based on their distance to control cells. Instead of applying a fixed threshold for all TFs, however, we used the 80th percentile of the distance of control cells to their respective centroids. TF cells with an Euclidean distance to the centroid of control cells greater than this threshold were considered as “functional”, indicating a substantial transcriptomic shift, often driven by TF dose (**Supplementary Fig. 5c**), while those below it were labelled as “non-functional”. To mitigate dose variations and the potential interference arising from the ‘non-functional’ cells, we established a common threshold for all TFs. Through different iterations, we determined that the retention of ‘functional’ cells and exclusion of ‘non-functional’ ones is balanced at the 80th percentile of the distance between control cells to their centroids (**Supplementary Fig. 5b**, **d**, and **e**).

Supplementary Note 9: Logistic model and TF classification

To study and compare the regulatory potential among individual TFs, a self-starting nonlinear least squares logistic model was used to fit the TF-induced transcriptomic changes against the TF dose using the nls function from the package states (algorithm = ‘SSlogis’). As illustrated in **Supplementary Fig. 6**, we first filtered TFs based on the number of cells and the dose range for consistency and comparability across various TFs. TFs were excluded if they had fewer than 30 cells and either fewer than 3 cells in the low-dose bin (dose < 1.68) or a maximum dose below 3.5. We then applied the logistic model to a total of 270 TFs of which 36 were finally still excluded due to convergence failures. Within the model, we estimated three parameters: Asym, Xmid, and Scal, representing the asymptote, the TF dose at the point of inflection, and the inverse slope at the point of inflection, respectively. As the dose of certain TFs might not have been sufficient to reach saturation, their asymptote sometimes largely surpassed the maximum observed transcriptomic change. To limit potential overestimation, we used model-inferred transcriptomic changes at the maximum observed dose, defining this property as “TF reprogramming capacity”. High-capacity TFs were defined as TFs with maximum predicted transcriptomic changes equal or above 0.23, indicating a strong transcriptional effect. Within the high-capacity TF group, we then evaluated whether the dose sensitivity is either low or high, as defined

by the model-inferred transcriptomic response at the mean dose of all TFs. This resulted in the categorization of all TFs into three major groups: 32 high-capacity and high dose-sensitive, 44 high-capacity and low dose-sensitive, and 158 low-capacity TFs.

Supplementary Note 10: Analysis for intolerance to loss of function

The mutational constraints quantified from variation in 141,456 humans were downloaded from gnomAD[4,5]. A Fisher's exact test was applied to compare the pLI (probability of being loss-of-function intolerant) score and the LOEUF (loss-of-function observed / expected upper bound fraction) across human orthologs of high-capacity TFs and low-capacity TFs, by using a typical threshold of 0.9 for pLI and a gnomAD-suggested threshold of 0.6 for LOEUF, respectively.

Supplementary Note 11: Comparison of cell cycle dynamics across TFs

We compared the interaction between the cell cycle and all probed TFs. Since all TF cells were confluent, only confluent mCherry-expressing cells were used as control in this analysis to avoid the confounding effect from non-confluent mCherry-expressing cells on cell proliferation rate[6]. A Fisher's exact test was applied to compare the proportion of S-G2/M cells between TF and control cells (**Fig. 6b** and **Supplementary Fig. 7**). To go beyond simplified discrete phase classification, which overlooks the circular and continuous nature of the cell cycle, we compared the distributions of S or G2/M scores between TFs and control cells using a Wilcoxon rank sum test. However, the interdependence of S and G2/M scores, reflected by the significant correlation of the two scores in control cells, could introduce bias in the interpretation (**Supplementary Fig. 4b**). To address this, we quantified cell distribution throughout cell cycle progression by analysing the cell density along the S and G2/M scores (**Extended Data Fig. 8a-d**). A kernel density estimate and two-sample comparison tests were applied to infer and compare the two-dimensional (2D) density distributions of S and G2/M scores between TF and control cells using the kde.test function of ks (v1.14.1)[7]. All statistical tests in these analyses were followed by FDR correction. An adjusted P value < 0.05 was considered statistically significant.

Supplementary Note 12: Cell death staining

C3H10T1/2 cells were transduced with mCherry (control), *Cebpa*, and *Mycn* followed by Puromycin selection and 5 days of doxycycline-induced TF overexpression as described in **Supplementary Notes 1-3**. Cells were stained with fluorescence dyes: Propidium iodide 750 nM for viability and Hoechst 5 µg/mL for nuclei. Cells were incubated with dyes in PBS for 30 min in the dark, washed twice with PBS, and imaged.

Supplementary Note 13: Customized adipocyte gene set

Fabp4, *Lpl*, *Pparg*, *Lipe*, *Adipoq*, *Cd36*, *Plin4*, *Plin2*, *Plin1*, *Cebpa*, *Cebpb*, *Cidec*, and *Cidea*

Supplementary Note 14: Housekeeping gene list for expression normalization

Actb, Gapdh, Ppia, Ppib, Tubb5, Rpl13, Rpl13a, Rpl19, Ubc, Gusb, Ywhaz, Eef1a1, and *Pgk1*

Supplementary Note 15: Cell barcode assignment for TF pair overexpression

For the experiments containing both TF pair and single TF cells, it was necessary to computationally distinguish cells overexpressing a pair of TFs from those overexpressing a single TF. Cell barcodes were classified as TF pair- or a single TF-overexpressing cell through a two-step process. First, only cell barcodes detected in both the 10x and TF enrichment libraries were kept. Then, the two TF-IDs accumulating the most reads in the TF-ID-enriched library were aggregated as "main pseudo TF-ID". Cell barcodes with less than 5 reads of "main pseudo TF-ID" were filtered out. Knee point analysis (as described in **Supplementary Note 5**) was performed on this aggregated value to filter background cell barcodes. A second knee point analysis was then performed on the number of reads of the main TF-ID (accumulating the most reads for each cell barcode) to distinguish singlets (above knee point threshold) from doublets and TF pair cells (below knee point threshold). By design, the TF-IDs used for the TF pair experiments were different from those in the single TF overexpression experiments. Consequently, cell barcodes below the threshold that did not display expected TF-ID combinations were filtered out as potential doublets. Among the remaining cell barcodes, cells were stratified according

to whether they overexpressed a single TF or TF pair based on whether both the first and second TF-ID were in our list of TF-ID combinations.

Supplementary Figures

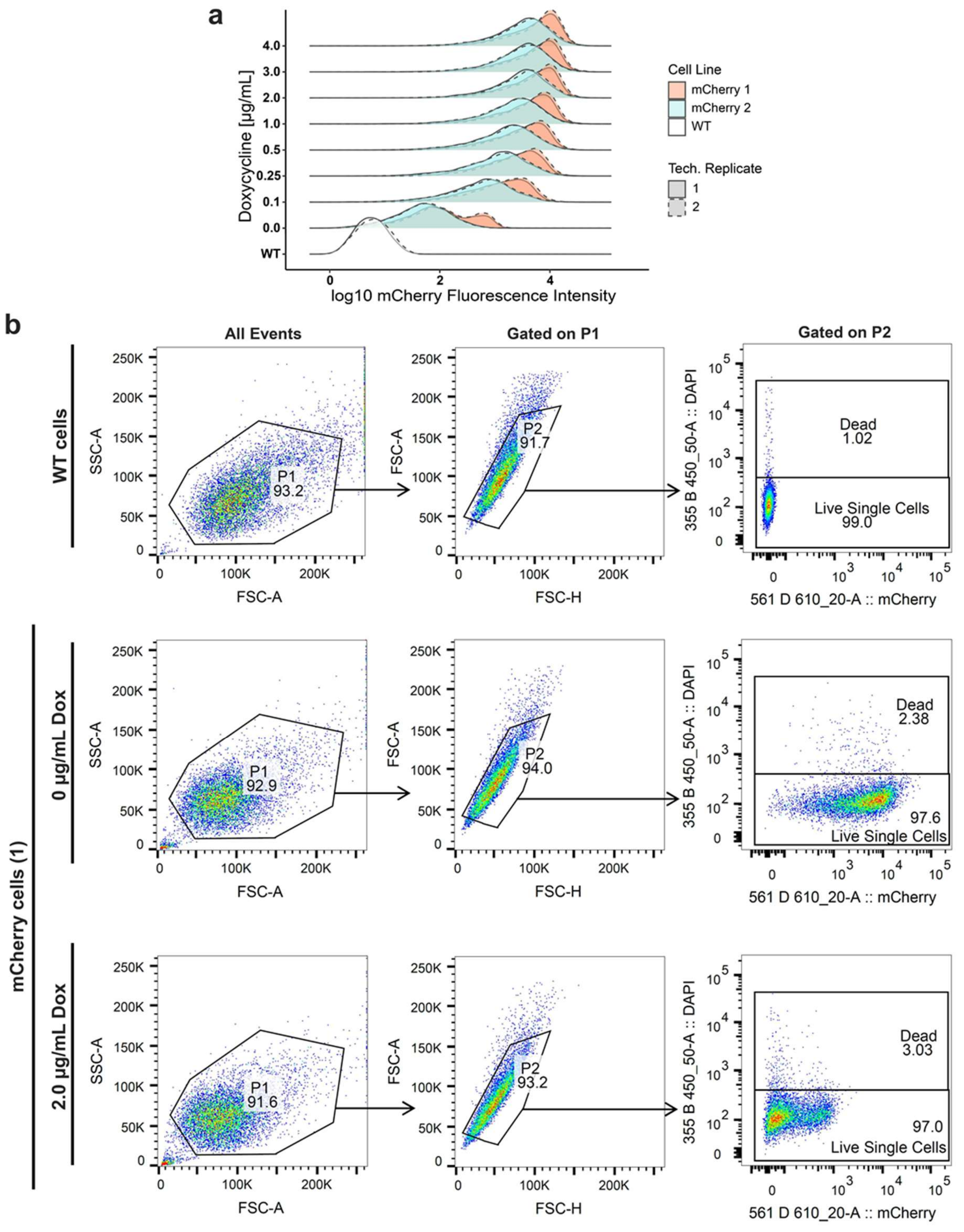

Supplementary Fig. 1: Doxycycline at 2 μg/ml induces ectopic expression near saturation level

a Ridgeline plot showing the distribution of mCherry fluorescence intensities of individual cells measured by flow cytometry across different doxycycline concentrations (0-4 μg/mL). Data were collected from two independent experiments, each with two independently generated mCherry cell lines. At least 8,000 cells were analyzed per cell line per experiment. WT refers to wildtype C3H10T1/2 cells.

b Representative pseudocolor plots illustrating all sequential gating strategies used to analyze mCherry fluorescence in live single cells. Cells were initially gated based on forward and side scatter (FSC vs. SSC) to exclude debris (P1), followed by gating on singlets (P2). Live single cells were selected based on exclusion of DAPI, and analyzed for mCherry fluorescence. Shown are representative plots from one experiment, including wildtype (WT) cells and one mCherry-expressing cell line, the latter uninduced and induced with doxycycline (0 μg/mL and 2.0 μg/mL Dox). The value within each gate indicates the percentage of the parent population.

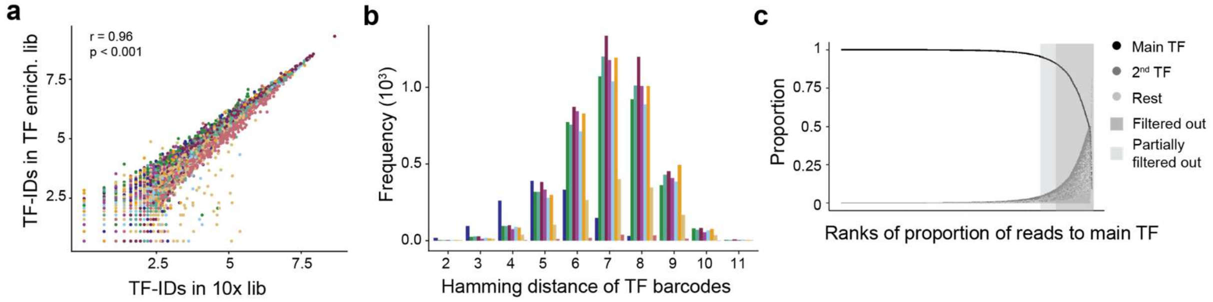

Supplementary Fig. 2: TF-ID detection and assignment

a Correlation between the log normalized values of reads mapping to the overexpression construct in the 10x libraries (10x lib) and in the TF enrichment libraries (TF enrich. lib). The Pearson correlation coefficient (r) and associated p value are shown.

b Bar plot showing the hamming distance of each pair of TF-ID barcodes and its frequency in each batch indicated by colors.

c Dot plot showing summarized results of TF assignment for all cells across all batches. The proportion of the most detected TF-ID (main TF), the second TF-ID (2nd TF), and the sum of the rest TF-IDs (rest) (calculated per batch) are plotted in decreasing order of proportion of their main TFs. The light and dark gray zones respectively mark cells partially (different threshold for each batch) and completely filtered out.

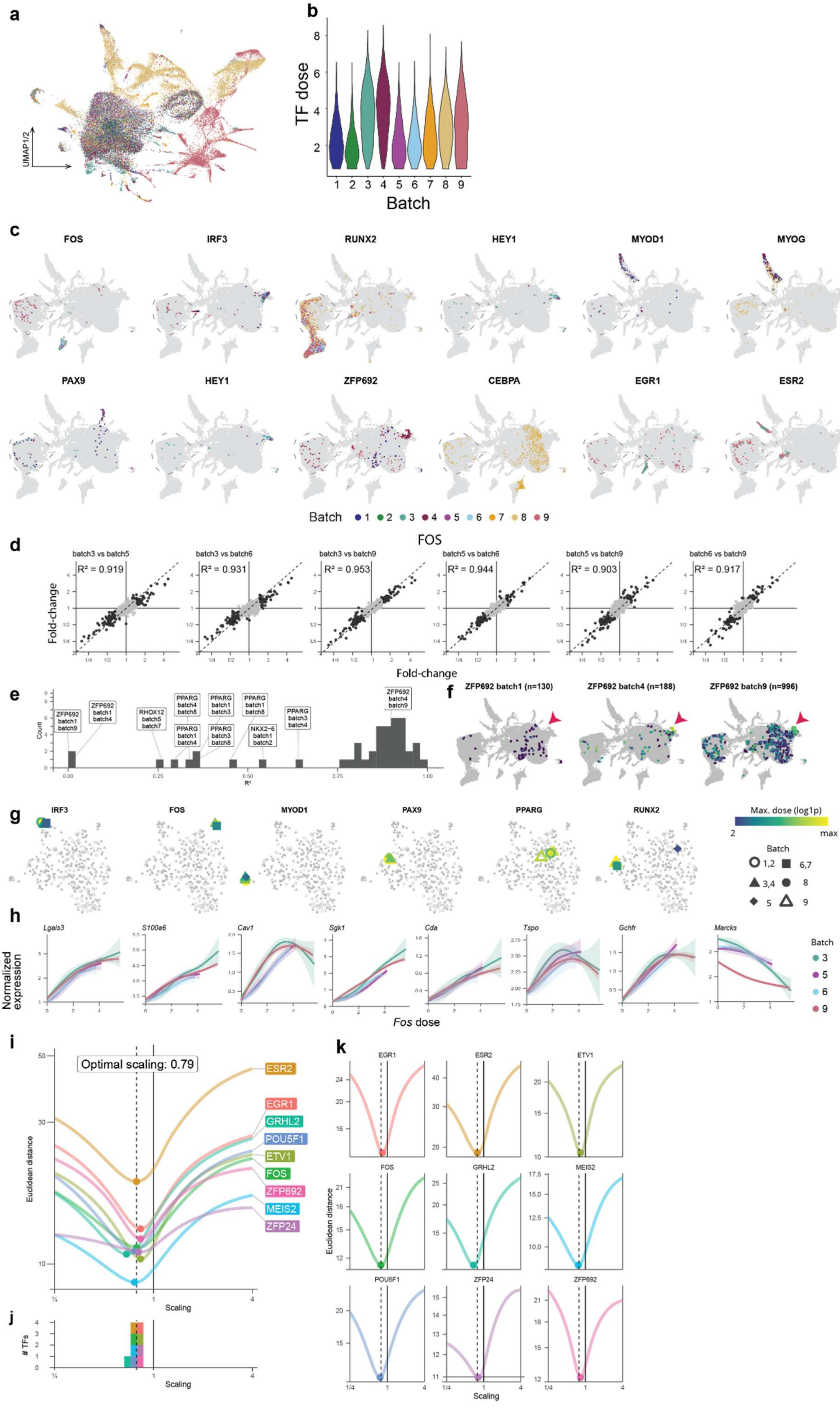
a
UMAP1/2
b
TF dose
Batch
1 2 3 4 5 6 7 8 9
c
FOS
IRF3
RUNX2
HEY1
MYOD1
MYOG
PAX9
HEY1
ZFP692
CEBPA
EGR1
ESR2
Batch 1 2 3 4 5 6 7 8 9
d
FOS
batch3 vs batch5
R² = 0.919
batch3 vs batch6
R² = 0.931
batch3 vs batch9
R² = 0.953
batch5 vs batch6
R² = 0.944
batch5 vs batch9
R² = 0.903
batch6 vs batch9
R² = 0.917
Fold-change
Fold-change
e
ZFP692 batch1 batch9
ZFP692 batch1 batch4
RHOX12 batch5 batch7
PPARG batch4 batch8
PPARG batch1 batch4
PPARG batch1 batch3
PPARG batch3 batch8
PPARG batch1 batch8
NKX2-6 batch1 batch2
PPARG batch3 batch4
ZFP692 batch4 batch9
Count
R²
f
ZFP692 batch1 (n=130)
ZFP692 batch4 (n=188)
ZFP692 batch9 (n=996)
g
IRF3
FOS
MYOD1
PAX9
PPARG
RUNX2
Max. dose (log1p)
2
max
Batch
1,2
6,7
3,4
8
5
9
h
Lgals3
S100a6
Cav1
Sgk1
Cda
Tspo
Gchfr
Marcks
Normalized expression
Fos dose
Batch
3
5
6
9
i
Optimal scaling: 0.79
ESR2
EGR1
GRHL2
POU5F1
ETV1
FOS
ZFP692
MEIS2
ZFP24
Euclidean distance
Scaling
j
TFs
Scaling
k
EGR1
ESR2
ETV1
FOS
GRHL2
MEIS2
POU5F1
ZFP24
ZFP692
Euclidean distance
Scaling

Supplementary Fig. 3: Comparison across batches

a UMAP plot of the batch-uncorrected TF atlas colored by experimental batches (see color legend in **c**).

b Violin plot showing the dose distribution of individual batches.

c UMAP plots of the TF atlas (grey) with cells from a particular TF highlighted, colored by batch.

d Fold changes for all genes between control and FOS cells for all pairs of batches. Shown are the corresponding R^2 values for the differentially expressed genes (in black).

e Distribution of R^2's of all high-capacity TFs shared between batches, with the TFs with the lowest R^2 highlighted.

f UMAP plots of the TF atlas colored by *Zfp692* dose across different batches. The red arrows highlight the main cluster of cells where the TF has a discernible effect (often because of high dose). The number in parentheses indicates the number of cells overexpressing the specific TF per batch.

g UMAP plots of pseudobulk expression for TFs that are replicated in at least 4 batches.

h Dose-response curves for the normalized expressions of the top eight differentially expressed genes modulated by FOS. Highlighted is the 95% percentage confidence interval as determined by GAM, fitted with a fixed smoothing parameter (sp=1) and restricted maximum likelihood (REML) for smoothing parameter estimation.

i Average Euclidean distance between dose response curves for all variable genes at different scaling parameters. The scaling factor scales the dose of batch 9 as to match the dose response curves of batch 3 and 4.

j Histogram of the most optimal scaling factor for all nine TFs.

k Same as **i** but for each individual TF.

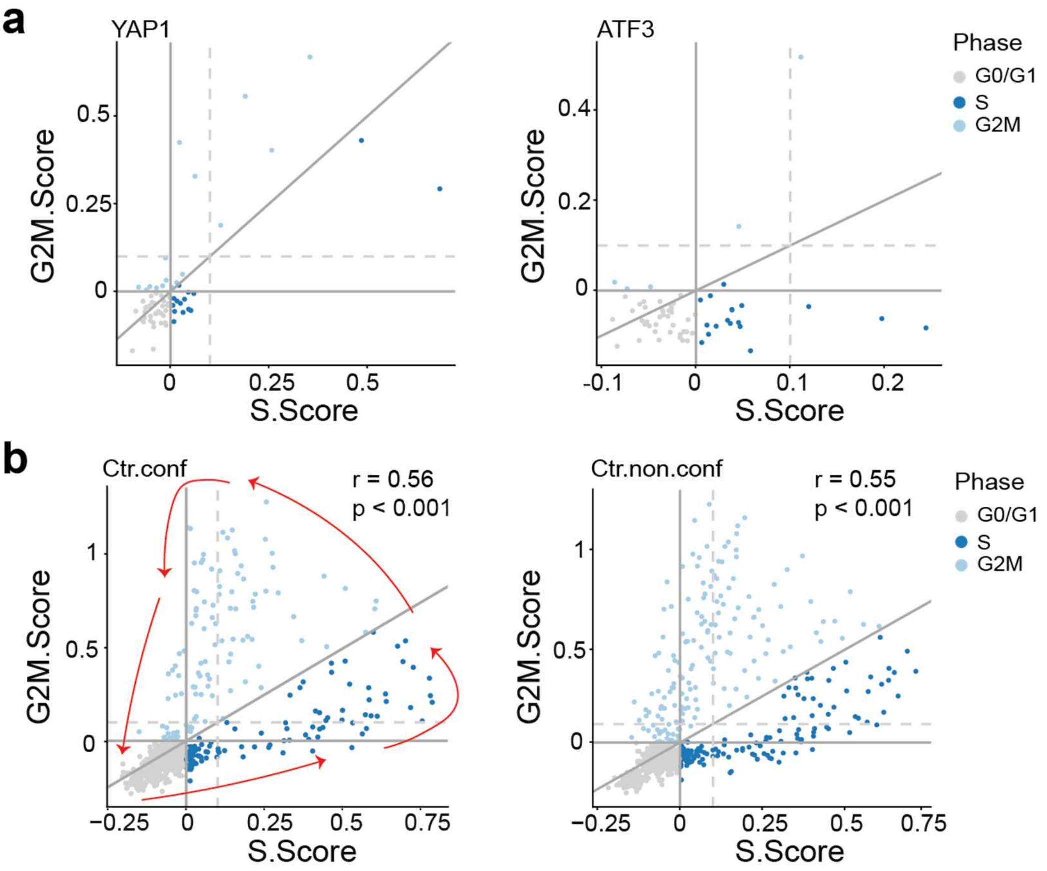

Supplementary Fig. 4: Examples of ambiguous cycling cells and correlations between S and G2M scores

a Scatter plots showing the correlation between S and G2M scores of two TF examples (YAP1 and ATF3), where the fraction of cells in each phase is strongly affected by the selected threshold (0 or 0.1, the latter is indicated by dashed gray lines). Cells are colored by the default phase.

b Scatter plots showing the correlation between S and G2/M scores in confluent (Ctr.conf, **left**) and non-confluent (Ctr.non.conf, **right**) control cells. Cells are colored by their default cell cycle phase. The Pearson correlation coefficient (r) and associated *P* value are shown. Red arrows on the left indicate the direction of cell cycle progression.

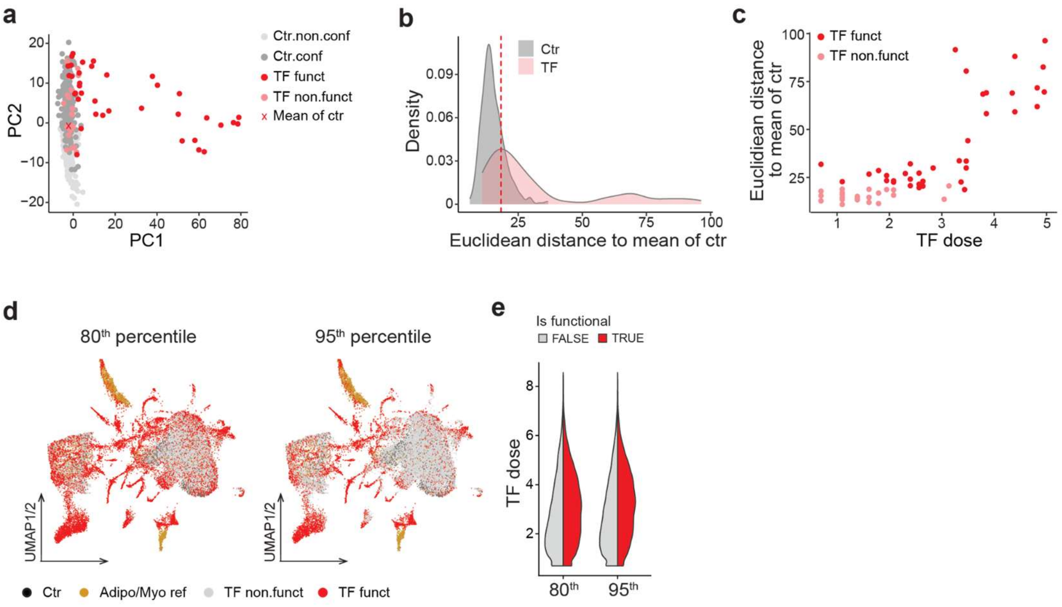

Supplementary Fig. 5: Identification of functional TF cells

a-**c** An example of identifying functional TF cells. See **Supplementary Note 8** for further details. (**a**) PCA analysis on CEBPA cells of one batch and their batch-paired control (ctr) mCherry cells. (**b**) Euclidean distance between each cell to the centroid of control cells in PCA space. Red dotted line represented 80% percentile of control cells distribution of euclidean distance to control centroid. (**c**) Euclidean distance of functional (red) and non-functional (pink) CEBPA cells was plotted over TF, here *Cebpa*, dose.

d UMAP plot of the TF atlas. Colors indicate control (ctr), adipose and myo reference (Adipo/Myo ref), functional (TF funct) and non-functional (TF non.funct) TF cells identified using the 80th percentile (**left**) or 95th percentile (**right**) as respective thresholds.

e Violin plots showing TF dose in functional (red) or non-functional (gray) cells of all TFs using the two thresholds (80th or 95th percentile in **d**).

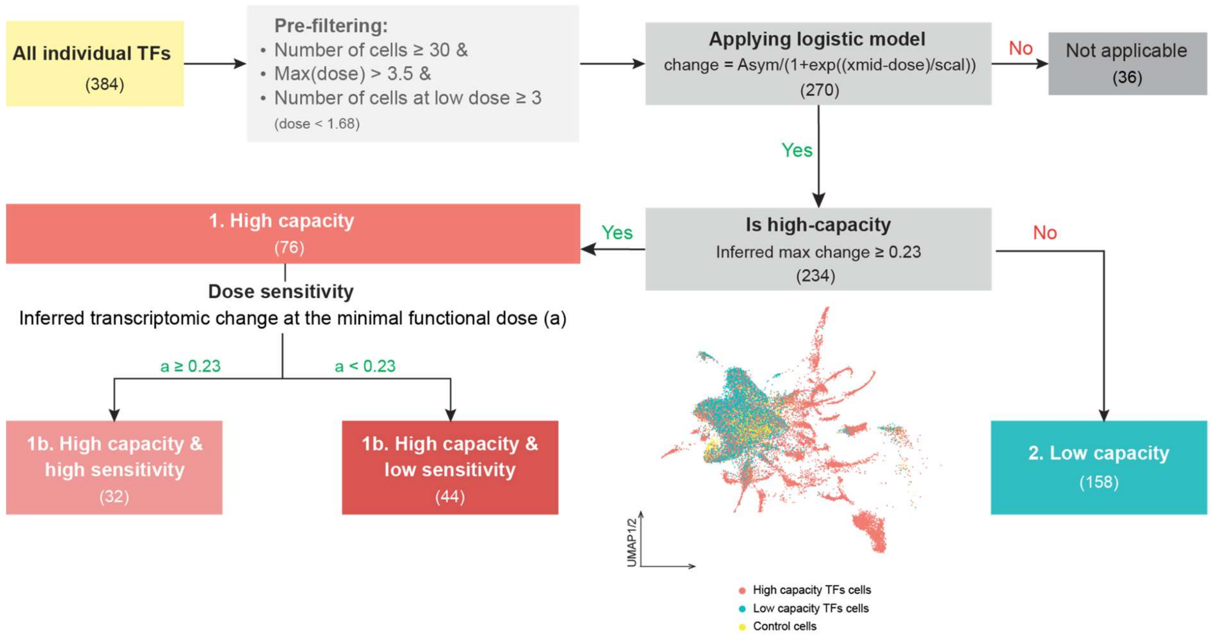

Supplementary Fig. 6: Flowchart of TF categorization

Flowchart showing TF categorization based on their reprogramming capacity and dose sensitivity by using the logistic regression model. As an inset, the UMAP plot of the TF atlas (shown in **Fig. 3a**) is colored by control, high-capacity and low-capacity TF cells.

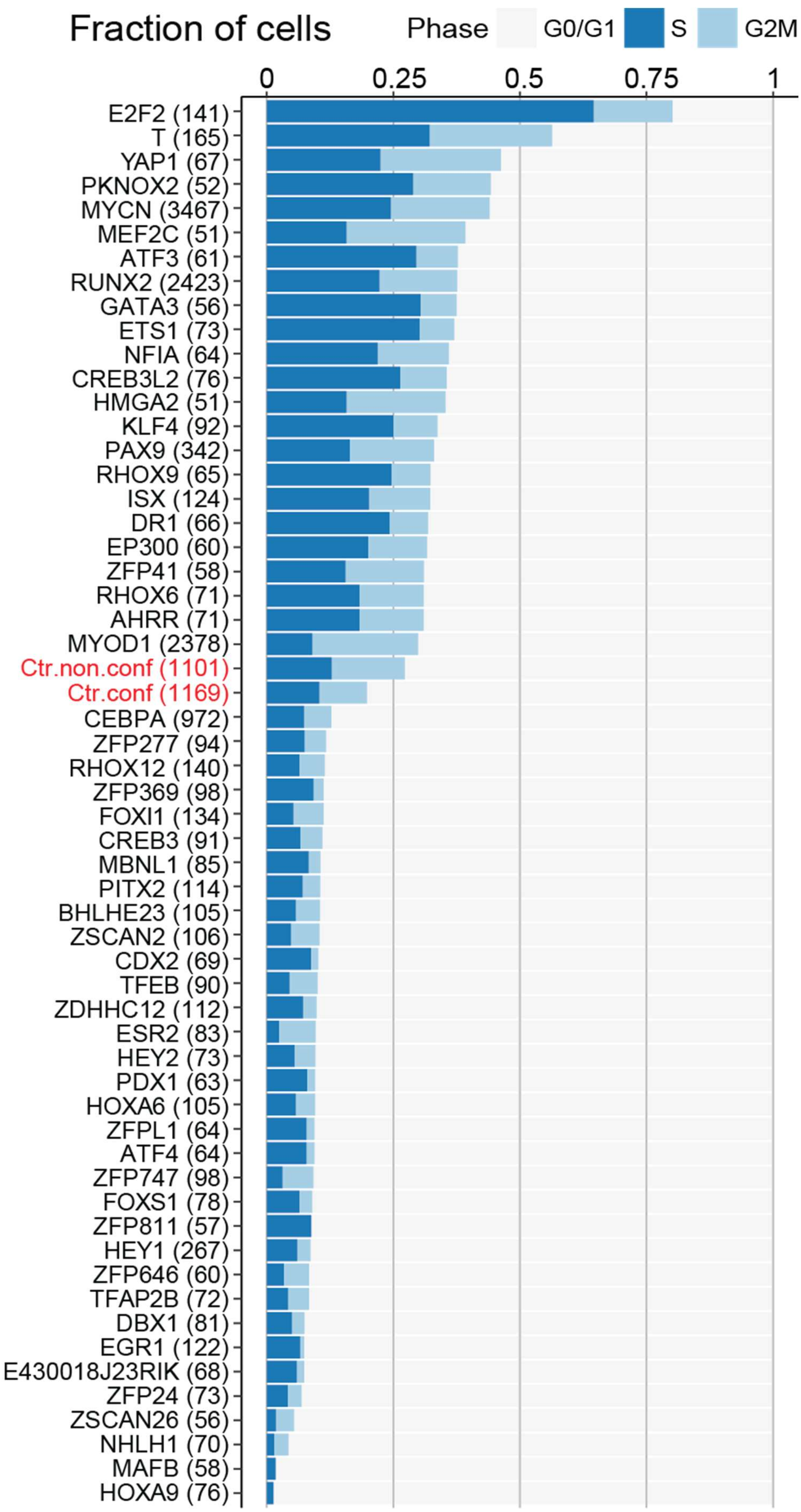
Fraction of cells
Phase
G0/G1
S
G2M
0
0.25
0.5
0.75
1
E2F2 (141)
T (165)
YAP1 (67)
PKNOX2 (52)
MYCN (3467)
MEF2C (51)
ATF3 (61)
RUNX2 (2423)
GATA3 (56)
ETS1 (73)
NFIA (64)
CREB3L2 (76)
HMGA2 (51)
KLF4 (92)
PAX9 (342)
RHOX9 (65)
ISX (124)
DR1 (66)
EP300 (60)
ZFP41 (58)
RHOX6 (71)
AHRR (71)
MYOD1 (2378)
Ctr.non.conf (1101)
Ctr.conf (1169)
CEBPA (972)
ZFP277 (94)
RHOX12 (140)
ZFP369 (98)
FOXI1 (134)
CREB3 (91)
MBNL1 (85)
PITX2 (114)
BHLHE23 (105)
ZSCAN2 (106)
CDX2 (69)
TFEB (90)
ZDHHC12 (112)
ESR2 (83)
HEY2 (73)
PDX1 (63)
HOXA6 (105)
ZFPL1 (64)
ATF4 (64)
ZFP747 (98)
FOXS1 (78)
ZFP811 (57)
HEY1 (267)
ZFP646 (60)
TFAP2B (72)
DBX1 (81)
EGR1 (122)
E430018J23RIK (68)
ZFP24 (73)
ZSCAN26 (56)
NHLH1 (70)
MAFB (58)
HOXA9 (76)

Supplementary Fig. 7: Fraction of cells in default cell cycle phase

Bar plot showing the fraction of cells in the cell cycle phase identified by using the default threshold of cell cycle scores from Seurat. The total number of cells is indicated in brackets. A Fisher's exact test followed by FDR correction was performed between confluent control (Ctr.conf) and each TF. In addition to Ctr.conf, only TFs and non-confluent control (Ctr.non.conf) that tested significantly (FDR-adjusted p-value < 0.05) are visualized here. Ctr.conf and Ctr.non.conf were highlighted in red.

References

1. Lamartina, S. *et al.* Construction of an rtTA2s-m2/ttskid-Based transcription regulatory switch that displays no basal activity, good inducibility, and high responsiveness to doxycycline in mice and Non-Human primates. *Mol. Ther.* **7**, 271–280 (2003).
2. Zare, H., Shooshtari, P., Gupta, A. & Brinkman, R. R. Data reduction for spectral clustering to analyze high throughput flow cytometry data. *BMC Bioinformatics* **11**, 403 (2010).
3. Butler, A., Hoffman, P., Smibert, P., Papalexi, E. & Satija, R. Integrating single-cell transcriptomic data across different conditions, technologies, and species. *Nat. Biotechnol.* **36**, 411–420 (2018).
4. Karczewski, K. J. *et al.* The mutational constraint spectrum quantified from variation in 141,456 humans. *Nature* **581**, 434–443 (2020).
5. Chen, S. *et al.* A genomic mutational constraint map using variation in 76,156 human genomes. *Nature* **625**, 92–100 (2024).
6. Gos, M. *et al.* Cellular quiescence induced by contact inhibition or serum withdrawal in C3H10T1/2 cells. *Cell Prolif.* **38**, 107–116 (2005).
7. Duong, T., Goud, B. & Schauer, K. Closed-form density-based framework for automatic detection of cellular morphology changes. *Proc. Natl. Acad. Sci.* **109**, 8382–8387 (2012).
