# Supplementary material for: Dissecting the impact of transcription factor dose on cell reprogramming heterogeneity using scTF-seq
Source: Nat Genet. 2025 Oct 3;57(10):2522–35. doi: 10.1038/s41588-025-02343-7 (PMC12513835; doi:10.1038/s41588-025-02343-7)
Supplement: Supplementary file 2 — Reporting Summary [file 41588_2025_2343_MOESM2_ESM.pdf]

nature portfolio

Corresponding author(s): Wanze Chen, Bart Deplancke

Last updated by author(s): Jun 10, 2025

Reporting Summary

Nature Portfolio wishes to improve the reproducibility of the work that we publish. This form provides structure for consistency and transparency in reporting. For further information on Nature Portfolio policies, see our Editorial Policies and the Editorial Policy Checklist.

Statistics

For all statistical analyses, confirm that the following items are present in the figure legend, table legend, main text, or Methods section.

n/a	Confirmed	
☐	☒	The exact sample size (*n*) for each experimental group/condition, given as a discrete number and unit of measurement
☐	☒	A statement on whether measurements were taken from distinct samples or whether the same sample was measured repeatedly
☐	☒	The statistical test(s) used AND whether they are one- or two-sided<br>*Only common tests should be described solely by name; describe more complex techniques in the Methods section.*
☐	☒	A description of all covariates tested
☐	☒	A description of any assumptions or corrections, such as tests of normality and adjustment for multiple comparisons
☐	☒	A full description of the statistical parameters including central tendency (e.g. means) or other basic estimates (e.g. regression coefficient) AND variation (e.g. standard deviation) or associated estimates of uncertainty (e.g. confidence intervals)
☐	☒	For null hypothesis testing, the test statistic (e.g. *F*, *t*, *r*) with confidence intervals, effect sizes, degrees of freedom and *P* value noted<br>*Give P values as exact values whenever suitable.*
☒	☐	For Bayesian analysis, information on the choice of priors and Markov chain Monte Carlo settings
☒	☐	For hierarchical and complex designs, identification of the appropriate level for tests and full reporting of outcomes
☐	☒	Estimates of effect sizes (e.g. Cohen's *d*, Pearson's *r*), indicating how they were calculated

Our web collection on statistics for biologists contains articles on many of the points above.

Software and code

Policy information about availability of computer code

Data collection	Basecalls performed on NextSeq 500/HiSeq 4000/NovaSeq 6000 result using bcl2fastq v.2.19/v2.20.
Data analysis	All source code used to analyze the data in this study can be found at https://github.com/DeplanckeLab/TF-seq and is publicly available upon publication.<br><br>R (v4.1.0, v4.3.1)<br>Cell Ranger (v3.0.2, v6.0.0, and v7.1.0 were used to generate count matrices of experiments 1-7, 8, and 9, respectively)<br>SamSPECTRAL (v1.46.0)<br>Seurat (v4.4.0)<br>scran (v1.22.1)<br>scanpy (v1.10.2)<br>clustree (v0.5.0)<br>cellpose (v3)<br>edgeR (v3.36.0)<br>fgsea (v1.18.0)<br>clusterProfiler (v4.0.5)<br>Hmisc (v5.1-1)<br>stats (v4.1.0)<br>Monocle3<br>ks (v1.14.1)

Flowjo v10.10

For manuscripts utilizing custom algorithms or software that are central to the research but not yet described in published literature, software must be made available to editors and reviewers. We strongly encourage code deposition in a community repository (e.g. GitHub). See the Nature Portfolio guidelines for submitting code & software for further information.

Data

Policy information about availability of data

All manuscripts must include a data availability statement. This statement should provide the following information, where applicable:

- Accession codes, unique identifiers, or web links for publicly available datasets
- A description of any restrictions on data availability
- For clinical datasets or third party data, please ensure that the statement adheres to our policy

All raw sequencing data generated in this paper has been deposited at ArrayExpress with the accession number E-MTAB-13010. Vector sequence for TF barcode counting has been deposited at github (vector_sequence, https://github.com/DeplanckeLab/TF-seq). The following genome assembly was used: GRCh38, release 96 from Ensembl. Marker genes of cell types of interest and hallmark gene sets were downloaded from MSigDB v2023.1.Mm and PanglaoDB v2019.
150 annotated single-cell datasets were downloaded from CELLXGENE census database. Mutational constraints quantified from variation in 141,456 humans were downloaded from gnomAD (v4.0). TF class annotations were collected from AnimalTFDB (v4.0). Microscopy data reported in this paper will be shared upon request.

Research involving human participants, their data, or biological material

Policy information about studies with human participants or human data. See also policy information about sex, gender (identity/presentation), and sexual orientation and race, ethnicity and racism.

Reporting on sex and gender	This information was not collected as only cell lines and no human or animal subjects were used.
Reporting on race, ethnicity, or other socially relevant groupings	This information was not collected as only cell lines and no human or animal subjects were used.
Population characteristics	This information was not collected as only cell lines and no human or animal subjects were used.
Recruitment	This information was not collected as only cell lines and no human or animal subjects were used.
Ethics oversight	This information was not collected as only cell lines and no human or animal subjects were used.

Note that full information on the approval of the study protocol must also be provided in the manuscript.

Field-specific reporting

Please select the one below that is the best fit for your research. If you are not sure, read the appropriate sections before making your selection.

☒ Life sciences ☐ Behavioural & social sciences ☐ Ecological, evolutionary & environmental sciences

For a reference copy of the document with all sections, see nature.com/documents/nr-reporting-summary-flat.pdf

Life sciences study design

All studies must disclose on these points even when the disclosure is negative.

Sample size	Nine independent scTF-seq experiements were performed with some TFs involved in more than one experiment as replicates (as described in the manuscript and metadata). 45978 cells covering 384 individual TFs and 7 TF pairs were used for the construction of scTF-seq atlas. Sample sizes for this study were determined based on established empirical thresholds commonly used in single-cell omics to ensure robust detection of biological signals and statistical reliability. For differential expression (DE) analysis, we required a minimum of 5 functional cells per TF to retain sufficient power to detect expression changes while mitigating false positives from underpowered comparisons. For gene set enrichment analysis (GSEA), which requires greater statistical resolution to assess pathway-level effects, we imposed a stricter threshold of ≥25 functional cells per TF. To evaluate TF dose sensitivity, reprogramming capacity, and cell fate transitioning—analyses that demand finer granularity to resolve dynamic or bifurcating behaviors—we restricted analysis to TFs with ≥30 cells. Cell cycle dynamics, which involve partitioning cells into discrete phases (G1/S/G2M), required ≥50 cells per TF to ensure adequate representation across phases for statistical testing. Statistic tests were performed for all the analyses as indicated in the Figure Legends and Methods accordingly.
Data exclusions	Single-cell RNA-seq samples showing low quality were excluded. The criteria are described in the Methods and Supplementary table 1. Cell clumps, debris, and 1% of outliers at the extreme lower or upper tails of the mean intensity distribution for individual RNAscope image channels were filtered out. The criteria are described in the Methods.
Replication	At least six transcription factors (as described in the metadata) were involved in more than one scTF-seq experiment. The imaging of mCherry expression was performed with three replicates. The profiling of mCherry fluorescence intensity was performed on two mCherry-

	overexpressing cell line, each with two replicates. RNAscope experiment was perfomed with at least two replicates. Validations of adipogenic capacity of single TFs or TF pairs were performed with at least three replicates. Cell death staining was performed with three replicates. These replicates successfully show consistent results.
Randomization	In this study, randomization was not performed due to the controlled experimental design and systematic nature of the scTF-seq approach. Specifically:<br>1. Only one isogenic cell line was used for the TF overexpression screen, minimizing genetic and environmental variability.<br>2. Each TF or TF pair was tested in a targeted manner to directly assess its effect, with cells assigned to experimental groups based on the TF(s) expressed (not random assignment). This ensures unambiguous attribution of observed outcomes to specific TFs.<br>3. Variability was addressed by performing nine independent scTF-seq experiments, with TFs tested across replicates. Batch effects were mitigated computationally (integration/batch correction) rather than via randomization.<br>4. Statistical robustness was ensured by excluding TFs with low cell counts (e.g., <5 or < 25 functional cells for DE or GSEA analysis), which serves a similar purpose to randomization in reducing noise from undersampled groups.<br>5. In single-cell perturbation screens, systematic testing of all factors under matched conditions (rather than randomized subsets) is standard practice to enable direct comparisons and atlas-scale profiling.
Blinding	The imaging of mCherry expression, lipid accumulation or cell viability, and RNAscope was performed blindingly. Blinding is not necessary for other analyses as they are quantitative and no subjective interpretation is required.

Reporting for specific materials, systems and methods

We require information from authors about some types of materials, experimental systems and methods used in many studies. Here, indicate whether each material, system or method listed is relevant to your study. If you are not sure if a list item applies to your research, read the appropriate section before selecting a response.

Materials & experimental systems

n/a	Involved in the study	
☒	☐	Antibodies
☐	☒	Eukaryotic cell lines
☒	☐	Palaeontology and archaeology
☒	☐	Animals and other organisms
☒	☐	Clinical data
☒	☐	Dual use research of concern
☒	☐	Plants

Methods

n/a	Involved in the study	
☒	☐	ChIP-seq
☐	☒	Flow cytometry
☒	☐	MRI-based neuroimaging

Eukaryotic cell lines

Policy information about cell lines and Sex and Gender in Research

Cell line source(s)	HEK293T and C3H10T1/2 cells used in this study were obtained from ATCC.
Authentication	None of the cell lines used were authenticated
Mycoplasma contamination	All cell lines used in this study were tested negative for mycoplasma contamination.
Commonly misidentified lines (See ICLAC register)	No cell lines is misidentified to our knowledge

Plants

Seed stocks	This information was not collected as only cell lines and no human or animal subjects were used.
Novel plant genotypes	This information was not collected as only cell lines and no human or animal subjects were used.
Authentication	This information was not collected as only cell lines and no human or animal subjects were used.

Flow Cytometry

Plots

Confirm that:

☒ The axis labels state the marker and fluorochrome used (e.g. CD4-FITC).

☒ The axis scales are clearly visible. Include numbers along axes only for bottom left plot of group (a 'group' is an analysis of identical markers).

☒ All plots are contour plots with outliers or pseudocolor plots.

☒ A numerical value for number of cells or percentage (with statistics) is provided.

Methodology

Sample preparation	C3H10T1/2 cells (Wildtype and mCherry overexpressing) were harvested by trypsinization, followed by quenching with growth medium. Cells were washed with PBS and resuspended in ice-cold PBS with DAPI (1 µg/mL) on ice.
Instrument	BD LSR Fortessa 5-laser cell analyzer
Software	Collection: BD FACSDiva 8.0.1<br>Analysis: FlowJo v10.10 and R v4.1.0
Cell population abundance	Between 75-90 % events passed the FSC/SSC gating used for analyzing single cells, of which at least 95 % were alive based on negative DAPI signal. Cells were not sorted further, just analyzed for mCherry fluorescence intensity.
Gating strategy	Cells were gated based on FSC and SSC to select single cells. Live cells were gated based on negative DAPI signal (355nm - 450/50nm). Live cells were analyzed for mCherry signal (561nm - 610/20nm).

☒ Tick this box to confirm that a figure exemplifying the gating strategy is provided in the Supplementary Information.
